# Select Abstracts From the 19th International Conference on Neonatal and Childhood Pulmonary Vascular Disease

**DOI:** 10.1002/pul2.70318

**Published:** 2026-06-05

March 19‐21, 2026

San Francisco, CA,

Jeffrey R. Fineman, MD

Department of Pediatrics

University of California, San Francisco

## 1 Placental Abnormalities of Malperfusion, Inflammation, and Meconium are Associated With Persistent Pulmonary Hypertension of the Newborn

Tsoi SM, Gasper C, Maltepe E, Chidboy M, Ozarslan N, Blavelt CA, Buarpung S, Cheung S, Steurer M, Keller RL, Fineman JR, Gaw SL.

University of California, San Francisco, Department of Pediatrics, Pathology and Laboratory Medicine, and Obstetrics, Gynecology & Reproductive Sciences. San Francisco, California

**Background/Hypothesis:** Persistent pulmonary hypertension of the newborn (PPHN) is a cause of neonatal hypoxic respiratory failure due to the failed transition of the pulmonary vasculature after birth. Mechanisms of disease are unknown, but we hypothesize they are directly related to insults in the intrauterine environment. The objective of this study was to elucidate whether placental histopathology representative of intrauterine insults during pulmonary vascular development are associated with a diagnosis of persistent pulmonary hypertension of the newborn (PPHN), and compare placental histopathologic lesions across PPHN etiologies.

**Materials and Methods:** We conducted a case‐control study of mother–infant dyads ≥ 35 weeks gestation who delivered at a tertiary care center between 2020–2025. Cases were infants diagnosed with PPHN and treated with inhaled nitric oxide; unaffected controls were infants without congenital anomalies. Placentas underwent blinded histopathologic review using standardized criteria. Multivariate logistic regression modeling was used to control for confounding maternal and infant factors.

**Results:** 106 placentas were analyzed (53 PPHN, 53 controls). Placental lesions were significantly more common in PPHN placentas, including fetal vascular malperfusion (30.2% vs 9.4%, p < 0.01), placental inflammation (66.0% vs 37.7%, p < 0.01), chronic presence of meconium (43.4% vs 15.2%, p < 0.01), and chorangiosis (7.6% vs 0%, p = 0.04). In adjusted analyses compared to controls, among 41 placentas with fetal development etiologies (e.g. congenital anomalies) of PPHN, fetal vascular malperfusion, placental inflammation and fetal inflammatory response were more common. Among 12 placentas with typical causes of PPHN (e.g. meconium aspiration syndrome), placental inflammation, maternal and fetal inflammatory responses, and meconium were more common.

**Conclusions:** PPHN placentas demonstrate lesions of malperfusion, inflammation, and chronic meconium, suggesting a complex interplay between intrauterine hypoxia and inflammation as a potential mechanism for the abnormal pulmonary vascular development and function seen in PPHN.

## 2 Pulmonary Hypertension‐Targeted Therapies in Adult and Pediatric Patients Supported With Ventricular Assist Devices: A Systematic Review

Rangu S^1^, Philip S^1^, Trivieri MG^2^, Glass L^1^, Chartan C^2^

^1^Texas Center for Pediatric and Congenital Heart Disease, UT Health Austin and Dell Children's Medical Center, Austin, Texas, USA, Icahn School of Medicine at Mount Sinai, New York, USA

**Background:** Pulmonary hypertension (PH) increases right ventricular (RV) afterload and contributes to morbidity and mortality in patients supported with ventricular assist devices (VADs). While adult literature has increasingly evaluated the role of PH‐targeted therapies in this context, pediatric literature remains sparse. Management of PH in VAD‐supported children remain highly variable, with no standardized approach to initiation, maintenance, or discontinuation of therapy, nor to screen for PH evolution during support. This systematic review synthesizes existing literature on the use of PH‐targeted therapies in adult and pediatric VAD patients to identify treatment patterns, outcomes, and evidence gaps that can guide future research and standardization of care.

**Materials and Methods:** We systematically reviewed PubMed/MEDLINE through August 2025 for studies on PH‐targeted therapies in adult and pediatric VAD patients. Eligible interventions included phosphodiesterase‐5 inhibitors, prostacyclins (PGI2), endothelin receptor antagonists (EREs), and inhaled nitric oxide (iNO). Outcomes included pulmonary hemodynamics, RV failure, transplant candidacy/success, survival, and adverse events.

**Results:** 16 studies met inclusion criteria (12 adult, 4 pediatric). Adult data demonstrates consistent short‐term hemodynamic benefit from perioperative sildenafil and iNO; with inhaled PGI2 have shown to be equivalent in randomized trials. Longer‐term PDE5 inhibitor therapy demonstrated benefit in small cohorts but not in registry analyses, where preoperative use was associated with increased bleeding and no survival benefit. EREs (including Macitentan) have shown promise in reducing pulmonary vascular resistance (PVR) in early studies. Pediatric data were limited to case reports and single‐center series. Sildenafil, bosentan, and prostacyclin facilitated reversal of elevated PVR, enabling successful transplantation in high‐risk children; one series reported 71% transplant success and 100% post‐transplant survival with PGI2 therapy.

**Conclusions:** PH‐targeted therapies play a critical role in adult and pediatric VAD support, improving perioperative hemodynamics and supporting transplant candidacy. Evidence strongly supports the use of sildenafil, PGI2, and iNO; pediatric findings are encouraging but require validation with multicenter prospective studies.

## 3 Monitoring Pulmonary Artery Pressure During Exercise in Pulmonary Arterial Hypertension Using the CardioMEMS Device: Initial Experience

Puyana Rodríguez JM^1*^, Toledano Navarro M^1^, Álvarez Fuente M^1^, Garrido‐Lestache Rodríguez‐Monte E^1^, Molina Borao I^1^, Rivero Jiménez N^1^, Barranco Fernández I^1^, Martín Pinacho JJ^2^, Torres Rubio P^2^, Izquierdo del Monte MG^1^, Del Cerro Marín MJ^1^

^1^Department of Pediatric Cardiology and Congenital Heart Disease, Ramón y Cajal University Hospital, Madrid, Spain, ^2^Department of Radiology, Ramón y Cajal University Hospital, Madrid, Spain

*The first author is a Pediatric Cardiology and Congenital Heart Disease fellow.

**Background**: CardioMEMS (CM) is an implantable wireless hemodynamic monitoring system approved for adults with heart failure that enables remote measurement of pulmonary artery pressure (PAP). Experience in pediatric pulmonary hypertension (PH) is limited, particularly regarding PAP monitoring during exercise.

**Materials and Methods**: This single‐center retrospective descriptive study included children and adolescents with PH who underwent cardiopulmonary exercise testing (CPET) after CM implantation.

**Results**: Between April 2025 and January 2026, eight patients were evaluated for CM implantation; the procedure was not performed in two due to unfavorable pulmonary vascular anatomy. Implantation under conscious sedation was successful in six patients (50% male), aged 13–20 years (mean 16.8 years). Diagnoses included congenital heart disease–associated PH (CHD‐PH, n = 4) and familial pulmonary arterial hypertension (FPAH, n = 2; TBX4 and AQP1 variants), with a median age at PH diagnosis of 6,3 years.

Targeted therapy at implantation consisted of quadruple therapy (iPDE + ERA + subcutaneous treprostinil + sotatercept) in three patients, triple therapy (iPDE + ERA + subcutaneous treprostinil) in one, and triple therapy (iPDE + ERA + sotatercept) in one. One patient with mixed PH (groups I and II) was not receiving PH‐specific therapy.

No procedure‐ or device‐related adverse events occurred during 6 months of follow‐up. CPET was performed a median of 3,3 months after implantation in three patients. PAP measurements were successfully obtained throughout exercise in all cases. In one patient, CPET with CM monitoring was performed before and 6 months after initiation of sotatercept therapy. Cardiac MRI after implantation was performed in five patients.

**Conclusions**: CM enabled continuous PAP assessment during CPET and provided clinically useful information on exercise tolerance and response to therapy. It may help guide individualized exercise recommendations and rehabilitation programs.

## 4 Controlled Interatrial Shunting With the Atrial Flow Regulator in Severe Pulmonary Arterial Hypertension: Hemodynamic and Clinical Results From the AFR‐Prophet Study (Poster Presentation)

Pattathu J, Bartunek J, Fiori E, Kopec G, Kilic T, Haas NA

Cardiovascular Center Azorg, Aalst, Belgium; Ludwig Maximilian University Munich, Munich, Germany; and participating centers in Poland and Turkey

**Background/Hypothesis:** In advanced pulmonary arterial hypertension, right ventricular failure and reduced transpulmonary flow result in impaired left ventricular filling and low cardiac output. Balloon atrial septostomy may improve symptoms but is limited by unpredictable shunt size and spontaneous closure. We hypothesized that a controlled and durable interatrial shunt using the atrial flow regulator would improve hemodynamics and right ventricular performance in high‐risk patients.

**Materials and Methods:** AFR‐Prophet is a prospective, multicenter study enrolling patients with severe pulmonary arterial hypertension despite optimized therapy. Twenty‐five patients underwent the procedure; 24 received the implant. The primary endpoint was serious adverse device‐ or procedure‐related events at 90 days. Secondary endpoints included changes in hemodynamic, echocardiographic, and functional parameters at 3 months and clinical outcomes at 1 year.

**Results:** At 3 months, pulmonary vascular resistance decreased and cardiac index increased significantly despite a modest reduction in arterial oxygen saturation. Mixed venous saturation improved, consistent with enhanced systemic blood flow.

Echocardiography demonstrated reduced right ventricular end‐diastolic diameter and right‐to‐left ventricular ratio, improved tricuspid annular plane systolic excursion, and better right ventricular–pulmonary artery coupling. N‐terminal pro‐brain natriuretic peptide levels decreased, 6‐minute walk distance increased, and 66% of patients improved by at least one New York Heart Association functional class. At 90 days, 28% experienced serious adverse events. During 1‐year follow‐up, 9 patients died and 3 underwent lung transplantation.

**Conclusions:** Controlled interatrial shunting with the atrial flow regulator improves right ventricular unloading, cardiac output, and functional status in carefully selected patients with severe pulmonary arterial hypertension. These findings support further evaluation of earlier application and optimized shunt sizing strategies.

## 5 From Empiric Vasodilation to Physiology‐Guided Care: A Decade of Evolving Pulmonary Hypertension Management in Congenital Diaphragmatic Hernia

Wanamaker, S.K.; Suttles, T.L.; Thompson, M.T.; Schuman‐McCoy, J.M.; Chen, Y.J.; Pont, M.M.; Wilkinson, M.H.; Chartan, C.A.

Dell Children's Medical Center, Austin, TX

**Background/Hypothesis:** Growing recognition of the complex ventricular‐pulmonary interactions in congenital diaphragmatic hernia (CDH) has led to increasing caution against empiric early pulmonary vasodilator therapy without characterization of the underlying cardiac phenotype. We sought to examine temporal changes in pulmonary hypertension management in infants with CDH over the past decade and to assess whether evolving practice patterns coincided with changes in clinical outcomes.

**Methods and Methods:** Infants recorded in the CDH Study Group Registry between 2015 and 2024 who were inborn or transferred to a reporting center within 30 days of life were included. Patients without an echocardiogram within the first three days of life or without documented ventricular function were excluded. Admissions from 2015–2019 were categorized as Era 1 (E1) and from 2020–2024 as Era 2 (E2). Demographics, ventricular function, pulmonary hypertension–directed therapies, and clinical outcomes were compared between eras.

**Results:** A total of 4,092 infants met inclusion criteria, with similar baseline characteristics between eras (Table 1). CDH repair rates increased from 88.4% in E1 to 91.9% in E2 (p < 0.001). Mortality declined from 24.5% to 20.1% (p = 0.001), and ECMO utilization decreased from 29.8% to 25.1% (p < 0.001). Use of inhaled nitric oxide decreased significantly from 61.2% in E1 to 51.6% in E2 (p < 0.001), with a marked reduction in early initiation on days 0–1 of life (76.4% vs. 62.0%, p < 0.001) (Table 2). Use of milrinone and phosphodiesterase‐5 inhibitors did not differ significantly between eras.

**Conclusions:** Over the past decade, pulmonary hypertension management in CDH has shifted away from early empiric pulmonary vasodilation. This evolution in practice coincided with improved survival and reduced ECMO utilization, suggesting that refined cardiopulmonary assessment ‐ rather than broader early therapy ‐ may be central to improving outcomes in CDH.

Table 1. Patient Characteristics.

E1 represents patients registered between 2014‐2019, and E2 represents patients registered between 2020‐2024. Defect types C and D are large defects, as defined by the CDH Study Group staging system. Categorical variables are represented in percentages and p‐values generated using Pearson's Chi‐square test. Continuous variables are represented in mean and standard deviation and p‐value generated using Student's t‐test.

*statistically significant

**Table:** 

	Total (n = 4092)	Missing (n)	E1 (n = 2357)	E2 (n = 1735)	p‐value
**Female (%)**	1745	29	1002	743	0.62
	(42.9%)		(42.6%)	(43.4%)	
**Estimated Gestational Age (weeks)**	37.5 (+/‐	12	37.5 (+/‐	37.5 (+/‐	0.37
	2.1)		2.2)	2.1)	
**Birth Weight (kg)**	2.97 (+/‐	16	2.96 (+/‐	2.97 (+/‐	0.47
	0.61)		0.61)	0.60)	
**1 minute APGAR**	4.9 ( + /− 2.5)	156	4.9 (+/‐	5.0 (+/‐	0.25
			2.5)	2.5)	
**5 minute APGAR**	6.7 ( + /− 2.1)	186	6.7 (+/‐	6.8 (+/‐	0.23
			2.1)	2.1)	
**Defect Type C or D (%)**	1867	460	1051	816	0.46
	(51.4%)		(50.9%)	(52.1%)	
**Repaired (%)**	3676	2	2083	1593	< 0.001*
	(89.9%)		(88.4%)	(91.9%)	
**Mortality (%)**	925	8	577	348	0.001*
	(22.6%)		(24.5%)	(20.1%)	
**ECMO Utilization (%)**	1134	12	701	433	< 0.001*
	(27.8%)		(29.8%)	(25.1%)	
**RV dysfunction (%)**	829	0	388	441	< 0.001*
	(20.3%)		(16.5%)	(25.4%)	
**LV dysfunction (%)**	336 (8.2%)	0	128 (5.4%)	208 (12%)	< 0.001*
**Biventricular dysfunction (%)**	688	0	413	275	0.16
	(16.8%)		(17.5%)	(15.9%)	

**Table 2. Practice Pattern Changes in Medical Management of Pulmonary Hypertension.** The number of patients exposed to therapy are represented in percentages and p‐values generated using Pearson's Chi‐square test. E1 represents patients registered between 2014‐2019, and E2 represents patients registered between 2020‐2024. PDE5‐i group includes intravenous and enteral sildenafil and tadalafil. iNO= inhaled nitric oxide. PDE5‐i= phosphodiesterase‐5‐inhibitor.

*statistically significant.

**Table:** 

		Total	E1	E2	p‐value
**iNO**	**Patients exposed (%)**	2340	1444	896	< 0.001*
		(57.2%)	(61.3%)	(51.6%)	
	**Exposed on day 0‐1 (%)**	1638	1088	550	< 0.001*
		(70.9%)	(76.4%)	(62.0%)	
**Milrinone**	**Patients exposed (%)**	1684 (41%)	941 (39.9%)	743	0.06
				(0.43%)	
	**Exposed on day 0‐1 (%)**	1056	604 (64.7%)	452	0.19
		(63.3%)		(61.6%)	
**PDE5‐i**	**Patients exposed (%)**	1406	827 (35.1%)	579	0.25
		(34.6%)		(33.3%)	

## 6 Title: Transition From Sildenafil to Tadalafil for Pulmonary Hypertension in Infants and Young Children: A Single‐Center Retrospective Experience

Musembi L^1^, Champion C^1^, Dryzer S^1^, Chartan C^1^

Dell Children's Medical Center, Center for Pulmonary Hypertension, The University of Texas at Austin, Dell Medical School, Austin, Texas^1^

**Background:** Phosphodiesterase‐5 inhibitors are commonly used in infants and young children with pulmonary hypertension (PH). Sildenafil requires multiple daily dosing and may be limited by adherence or gastrointestinal intolerance. Tadalafil offers once‐daily dosing; however, data describing echocardiographic response and safety in children under two years of age remain limited.

**Methods:** We performed a single‐center retrospective review of infants and children < 2 years of age with PH who transitioned from sildenafil to tadalafil. Patients were included if they had at least one echocardiogram ≤ 30 days prior to tadalafil initiation and one echocardiogram 1–120 days after initiation, each with a measurable tricuspid regurgitation jet (TRJ). Echocardiographic parameters included TRJ‐derived right ventricular systolic pressure (RVSP) estimates and qualitative right ventricular function. Demographics, World Symposium on Pulmonary Hypertension (WSPH/WHO) group, Panama classification, NYHA functional class (when documented), dosing, and adverse events were abstracted. Paired pre‐ and post‐transition echocardiographic findings were compared descriptively.

**Results:** Thirty patients met inclusion criteria. Median age at transition was < 12 months, with the majority classified as WSPH Group 3 or Group 1 disease. All patients had paired echocardiograms with measurable TRJ. Following transition to tadalafil, TRJ‐derived RVSP estimates were **stable or improved in the majority of patients**, with no signal for systematic worsening. Qualitative right ventricular function was unchanged or improved in most cases. Where documented, NYHA or Panama functional class remained stable or improved. Tadalafil was well tolerated; no patients discontinued therapy due to hypotension or clinically significant adverse effects. Caregivers frequently cited improved ease of administration with once‐daily dosing.

**Conclusions:** In this single‐center cohort of infants and young children with pulmonary hypertension, transition from sildenafil to tadalafil was associated with **stable echocardiographic hemodynamics and good short‐term safety**, with potential benefits in medication adherence. These findings support tadalafil as a reasonable alternative to sildenafil in selected patients under two years of age.

## 7 Physical Activity as a Vital Sign in Pediatric Pulmonary Hypertension

Walton MM^1^, Watson JS^1^, Holbert J^1^, Desai L^2^, McIntosh A^1^, White, DA^1,^ Jensen M^1^.

^1^Ward Family Heart Center, Children's Mercy Kansas City, School of Medicine, University of Missouri Kansas City, Kansas City, MO, ^2^Texas Children's Heart Center, Texas Children's Hospital, Baylor College of Medicine, Houston, TX

**Background/Hypothesis:** Children with pulmonary hypertension frequently experience reduced exercise capacity and activity intolerance due to impaired right ventricular function and limited cardiovascular reserve. Routine assessment of physical activity is not standardized in pediatric cardiology practice. This study hypothesized that integrating an assessment of physical activity into outpatient workflow would be feasible and would align with clinical indicators of disease severity in pediatric patients with pulmonary hypertension.

**Materials and Methods:** A retrospective and prospective review of physical activity was performed via surveys collected through a standardized digital platform (REDCap) and administered prior to cardiology visits for patients 4 years of age and older. Surveys were linked with clinical data including cardiac diagnoses, functional class, echocardiographic findings, exercise tests, six‐minute walk test, medication profiles, and documented activity limitations.

Descriptive analysis was used to characterize reported daily activity patterns, and the relationship to clinical status was assessed.

**Results:** Data were available (34 surveys from 14 unique patients) for children aged 4 to 19 years with pulmonary hypertension across World Health Organization groups 1 through 3. Most patients reported fewer than 60 minutes per day of active play or exercise. Participation in organized physical activity was uncommon, while unstructured play was more frequently reported. Children with advanced functional class, Eisenmenger physiology, or moderate to severe right ventricular dysfunction reported the lowest activity levels and more frequent activity limitations. Reported activity patterns corresponded with clinical markers of disease severity, including reduced six‐minute walk distance, decreased right ventricular systolic functional indices, and higher estimated right ventricular systolic pressure.

**Conclusions:** Assessment of physical activity as a vital sign in pediatric pulmonary hypertension is feasible and provides clinically meaningful information about real‑world activity behavior. The trend of reported activity levels aligns with functional status and objective exercise capacity. Incorporation of this measure into standard practice may support individualized counseling, longitudinal monitoring, and development of targeted activity interventions for children with pulmonary hypertension.

## 8 Streamlining Pediatric Pulmonary Hypertension Care: The Impact of Telemedicine on New Referrals

Merrill KM, Davis A, Mowbray W, Jackson EO, Riker M, Yung D. Seattle Children's

Hospital and University of Washington, Seattle, WA

**Background:** New patients referred to a pulmonary hypertension comprehensive care center (PHCC) generally want to establish care in a timely manner while minimizing travel burden.

Outpatient evaluation often requires coordination of multiple diagnostic studies which can require several weeks' lead time. While telehealth expanded in 2020 during the COVID‐19 pandemic, its role as a triage tool remains underexplored. Our center implemented telemedicine to expedite initial evaluation and reduce travel burden. The aim of this study was to compare outcomes of initial telemedicine versus in‐person consultation.

**Methods:** We reviewed records of new outpatient referrals since 2020, collecting demographics, referring specialty, home zip code, and pulmonary hypertension (PH) diagnosis.

**Figure 1:**
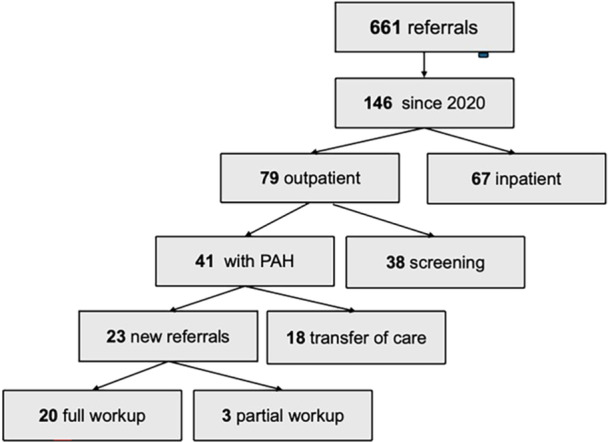
Participant flow diagram.

Distance to the center was calculated using zip codes. See Figure 1 for patient flow diagram.

**Results:** There were 79 new outpatients seen between 2020–2025 with 44 (56%) in‐person and 35 (44%) via telemedicine. Demographics are shown in Table 1. Patients seen via telemedicine more often lived > 100 miles from the PHCC, were referred by pediatric cardiologists, and had congenital heart disease (Table 2). Among patients referred for comprehensive PH evaluation, only 10 of 41 (24%) were evaluated in‐person, compared with 34 of 38 (90%) referred for PH screening. Of the 41 patients referred for PH evaluation, all 17 who lived > 100 miles were seen via telemedicine. Median (IQR) time from referral to initial visit for PH evaluation was 56 (27,116) days for 10 in‐person visits and 27 (8,42) days for 31 telemedicine visits.

**Discussion/Conclusion:** Telemedicine was predominantly utilized by patients undergoing comprehensive PH evaluation and residing > 100 miles from the PHCC. Telemedicine patients were seen sooner than in‐person. Telemedicine may help address geographic barriers and improve equity, potentially accelerating care while reducing travel burden. Future research will assess impact on diagnostic and treatment timelines, and patient‐centered outcomes.

Table 1. Patient demographics.

**Table:** 

		Total, n = 79	In‐person, n = 44	Telemedicine, n = 35
Year				
	2020	6	1	5
	2021	16	11	5
	2022	10	4	6
	2023	19	11	8
	2024	13	7	6
	2025	15	10	5
Age, years	mean	4.5	4.7	4.3
	SD	6.4	6.0	6.9
	median	8.2	8.3	7.9
	min	0.1	0.3	0.1
	max	20.5	17.3	20.5
Sex	male	36	21	15
	female	43	23	20
Race	White	41	21	20
	Asian	8	5	3
	White + (Asian, Mexican, Black, Filipino, American Indian)	8	5	3
	Black or African American	4	3	1
	American Indian	2	0	2
	Alaska Native	2	0	2
	Asian Indian	1	1	0
	Pacific Islander	1	1	0
	Mexican/Mexican American	1	0	1
	Bamar/Burman/Burmese	1	0	1
	Other	8	6	2
	Patient Declined	2	2	0
Ethnicity	Non‐Hispanic	68	37	31
	Hispanic	7	5	2
	Unknown	4	2	2

Table 2. Patient location, referrer specialty, and PH diagnosis.

**Table:** 

		Total, n = 79	In‐person, n = 44	Telemedicine, n = 35
Patient location from center, miles				
	0‐20	22	19	3
	20‐50	14	7	7
	50‐100	20	14	6
	100‐200	5	0	5
	200‐500	11	3	8
	500‐1000	2	0	2
	> 1000	5	1	4
Referral specialty	Pediatric Cardiology	39	9	30
	Other	13	12	1
	PCP	13	12	1
	Pediatric Pulmonary	11	9	2
	Pediatric Surgery	2	2	0
	PICU	1	0	1
PH screening	Yes	38	34	4
	No	41	10	31
PH diagnosis				
	No pulmonary hypertension after screening	20	17	3
	1.1 Idiopathic PAH	6	2	4
	1.2 Heritable PAH	9	7	2
	1.4.3 Portal hypertension	1	0	1
	1.4.4 Congenital heart disease	20	5	15
	1.5 PAH long‐term responders to calcium channel blockers	1	0	1
	1.7 Persistent PH of the newborn syndrome	3	3	0
	2.1 PH due to heart failure with preserved LVEF	1	0	1
	2.4 Congenital/acquired cardiovascular conditions leading to post‐capillary PH	2	1	1
	3 PH due to lung diseases and/or hypoxia	5	2	3
	3.5 Developmental lung disorders	9	5	4
	5 PH with unclear and/or multifactorial mechanisms	2	2	0

## 9 Intravenous/Subcutaneous Prostacyclin Initiation on Pediatric Acute Care Cardiology Units, a Mixed Methods Pediatric Acute Care Cardiology Collaborative Study on Practice Patterns and Short‐Term Outcomes

Miles KG^1^, Rodts M^1^, O'Neil M^1^, Gal D^2^, Schiller A^3^, Griffiths M^4^, Hopper RK^5^, Kipps AK^5^, Olive MK^3^, Pater C^1^, Sullivan RT^6^, Critser PJ^1^

^1^Cincinnati Children's Hospital Medical Center, University of Cincinnati, Cincinnati, OH, ^2^Children's Hospital Los Angeles, University of Southern California, Los Angeles, CA, ^3^C.S. Mott Children's Hospital, University of Michigan, Ann Arbor, MI, ^4^Children's Health Hospital, UT Southwestern University, Dallas, TX, ^5^Lucile Packard Children's Hospital, Stanford University, Palo Alto, CA, ^6^Monroe Carell Jr. Children's Hospital, Vanderbilt University, Nashville, TN

**Background:** Initiation of prostacyclin therapy, a cornerstone of pediatric PH, is frequently restricted to ICU settings resulting in significant resource utilization. Safety and necessary systems of care to support initiation in acute care cardiology units (ACCUs) are not well defined.

**Methods:** This mixed‐methods study explored new intravenous (IV)/subcutaneous (SQ) prostacyclin initiation on the ACCU utilizing registry data from the Pediatric Acute Care Cardiology Collaborative (PAC^3^). An additional survey explored center‐specific practices, strategies/barriers to ACCU prostacyclin initiation, and policies/protocols.

**Results:** Of > 145,000 encounters (10/2021‐1/2025), IV/SQ prostacyclin was initiated in 29 patients at 11 centers. Most were at PPHNet/PHA centers (21/29 encounters; 6/11 centers) involving patients with PAH (IPAH/HPAH, n = 13; PAH‐CHD, n = 6) or single ventricle CHD (n = 6). Three (10.3%) patients underwent Glenn palliation or heart transplantation after initiation of IV/SQ therapy, 4 (13.7%) patients required cardiac ICU transfer, and there were no cardiac arrests or mortalities. Of 32 survey responders (64% response rate), 7 (21.9%) centers initiate IV/SQ prostacyclin therapy on the ACCU (PPHNet/PHA centers=5/7). Criteria for ACCU initiation included outpatient stability (n = 4), reassuring echocardiogram (n = 2), and enteral/inhaled prostacyclin transition (n = 1). Support processes for ACCU initiation of IV/SQ prostacyclin (6/32 responders) included PH team support (n = 5), standardized protocols (n = 6), front‐line provider experience (n = 5), and nursing education (n = 6). Barriers (25/32 responders) included inadequate monitoring (n = 17), safety concerns (e.g nurse‐to‐patient ratio, n = 17), clinical status (n = 14), nursing education (n = 11), and limited PH team support (n = 10). Among nine IV/SQ prostacyclin initiation policies obtained, five were ACCU‐specific; Table 1 summarizes protocol commonalities.

**Conclusions:** IV/SQ prostacyclin initiation on the ACCU is rare but may be safe at PAC^3^ centers with appropriate protocols and processes. Identified supports, barriers, and protocol commonalities may help define the resources necessary to expand initiation of IV/SQ prostacyclin therapy on ACCUs.

Table 1. Protocol commonalities for IV/SQ prostacyclin therapy initiation and management on the ACCU.

**Table:** 

COMMON ELEMENTS OF AN ACCU INITIATION POLICY	
**Patient selection criteria**	Stable PH patients admitted from home or catheterization lab Stable or mild degree of right heart failure Primary PH team deems initiation and titration safe on ACCU
**Titration protocols**	Includes starting doses, titration intervals, dose‐adjustment increments, and therapeutic target. Treprostinil titration example: Start 1‐3ng/kg/min, ↑ 1‐4 ng/kg/min every 4‐24h to initial target dose (~20ng/kg/min, as per PH team) or as tolerated by side effects
**Order sets and flowsheets**	Prostacyclin order set (drug dose, drug concentration, order‐specific weight, and infusion rate). Printed flowsheet at bedside or in EPIC specifying prostacyclin pump administration specifics and titration plan
**Vital sign and side effect monitoring during titration**	Vital sign monitoring with initiation and titration (example: every 15 min. x4, every 30 min. x2, then Q4h) Review of common side effects Signs and management of acute drug discontinuation (ex: SQ site dislodgment) or bolus/overdose
**GENERAL CONSIDERATIONS FOR IV/SQ PROSTACYCLIN THERAPY ON ACCU**	
**Pump management**	Use of hospital (i.e., Medfusion 3500/4000) vs. home pump (ex: inpatient stay < 24h or family at bedside with home supplies). Step‐by‐step instructions for pump programming Tubing, filter set‐up (for IV infusion), syringe change frequency Use of central venous access for IV infusion Delineation of pump management (RN vs. family/patient) Transition to home pump (ex: specialty pharmacy RN or PH team within hours or day of discharge)
**SQ site management**	SQ site selection & dry site placement guidance Appropriate equipment with step‐by‐step instructions for site placement and dressing Medication pre‐treatment & supportive care (i.e., histamine antagonists, topical anesthetics, etc.) Monitoring SQ site (i.e., redness, swelling, drainage, or pain) Signs of compromised sites which may require exchange.
**General prostacyclin education**	Mechanism of action, drug half‐life, and indications for therapy Side effects, including vital sign monitoring (typically Q4h). Signs of acute drug discontinuation or bolus/overdose

## 10 Off‐Label Sotatercept Use in Pediatric Pulmonary Arterial Hypertension: A Single‐Center Retrospective Hemodynamic Study

Rao D, Haldeman S, Carroll J, Rao R.

University of Arizona, Tucson, Arizona; Rady Children's Hospital–San Diego, San Diego, California; University of California San Diego, San Diego, California

**Background/Hypothesis:** Pediatric pulmonary arterial hypertension remains a progressive and life‐threatening disease despite advances in vasodilator therapy. Sotatercept, an activin signaling inhibitor, has demonstrated disease‐modifying efficacy in adults by targeting pulmonary vascular remodeling rather than isolated vasoconstriction. Pediatric outcome data remain limited to ongoing clinical trials. We hypothesized that off‐label sotatercept use in children with advanced pulmonary arterial hypertension refractory to maximal medical therapy would be associated with favorable short‐term hemodynamic trends.

**Materials and Methods:** We conducted an institutional review board–approved retrospective review of pediatric patients with World Health Organization Group 1 pulmonary arterial hypertension treated with off‐label sotatercept at a single tertiary center. All patients were receiving maximal background triple therapy, including a phosphodiesterase‐5 inhibitor, endothelin receptor antagonist, and prostacyclin, yet demonstrated persistent symptoms and/or adverse hemodynamics. Right heart catheterization was performed prior to sotatercept initiation and approximately 24 weeks thereafter. Hemodynamic parameters, including pulmonary vascular resistance index, mean pulmonary artery pressure, and pulmonary capillary wedge pressure, were analyzed descriptively.

**Results:** Seven pediatric patients (median age 14 years, range 4–19) were included, representing idiopathic, heritable, and associated pulmonary arterial hypertension. Following sotatercept initiation, most patients demonstrated a reduction in pulmonary vascular resistance index and mean pulmonary artery pressure at approximately 24 weeks, with the greatest absolute improvements observed in those with the most severe baseline disease. Pulmonary capillary wedge pressure remained stable across all patients, suggesting that observed improvements were attributable to pulmonary vascular effects rather than changes in left‐sided filling pressures. Inter‐patient heterogeneity in response was observed.

**Conclusions:** In this highly selected pediatric cohort with advanced pulmonary arterial hypertension refractory to triple therapy, off‐label sotatercept use was associated with favorable short‐term hemodynamic trends without evidence of worsening left‐sided filling pressures. These findings provide early real‐world pediatric signal consistent with adult trial data and underscore the urgent need for prospective pediatric studies evaluating disease‐modifying therapies.

## 11 External Validation of a Multidimensional Classification for Neonatal Pulmonary Hypertension

Morgan, CE^1,*^, Levy, PT^2^, Mullen, MP^3^, Abman, SH^4^

^1^Department of Paediatric Cardiology, Great Ormond Street Hospital, London, UK, ^2^Division of Newborn Medicine, Boston Children's Hospital, and Department of Paediatrics Harvard Medical School, Boston, MA, USA, ^3^Department of Cardiology, Boston Children's Hospital, Boston, MA; Department of Paediatrics, Harvard Medical School, Boston, MA, USA, ^4^Department of Paediatrics and Paediatric Heart Lung Center, University of Colorado Anschutz Medical School and Children's Hospital Colorado, Aurora CO, USA

**Background/Hypothesis:** Neonatal pulmonary hypertension (PH) is a heterogeneous and time‐dependent condition that has been inadequately characterized by current static, etiology‐based classification systems, including the World Symposium on Pulmonary Hypertension classification. We propose a novel, multidimensional phenotyping framework integrating dominant physiology (P), etiologic context (E), and disease trajectory (T) to reflect longitudinal and dynamic changes in hemodynamics with bedside clinical reasoning.

This approach aligns classification with real‐world neonatal care and provides a foundation for improved risk stratification, precision‐based therapy, and longitudinal research in neonatal PH. We hypothesize that the “P–E–T” framework can be reproducibly applied by clinicians across disciplines with acceptable inter‐rater agreement and will demonstrate preliminary construct validity in representative neonatal PH cases.

**Materials and Methods:** Ten neonatal PH case vignettes were developed to represent distinct physiologic drivers (P), etiologic substrates (E), and temporal trajectories (T), including transitional maladaptation, bronchopulmonary dysplasia–associated PH, shunt‐mediated PH, and developmental pulmonary vascular disease. Case selection was structured to ensure representation across major P, E, and T categories, reducing prevalence imbalance and improving interpretability of κ‐based agreement estimates. Conference participants will classify cases via a QR‐linked electronic survey. Demographic data (specialty, training level, and self‐rated familiarity with neonatal PH) will be collected. We anticipate approximately 30 participants (≈300 ratings). Primary endpoints include inter‐rater agreement for P, E, and T using Fleiss' κ statistics and percent agreement. Secondary endpoints include completion time, usability ratings, and subgroup analyses by specialty and familiarity.

**Results:** We anticipate substantial agreement (κ ≥ 0.60) for clearly defined phenotypes, with lower agreement in mixed or evolving cases.

**Conclusions:** This external validation represents an initial step toward establishing reproducibility and clinical utility of a multidimensional neonatal PH classification, supporting improved risk stratification, therapeutic precision, and standardized characterization for future multicenter research.

## 12 Secretion of a Unique Human Placental Decoy Receptor Profile in Early‐Onset Preeclampsia: Implications for Pulmonary Vascular Developmental Injury

Pamidimukkla D^1,2*^, Pfau R^3^, Holtz AM^2^, Kotton DN^1,2,3^, Taglauer ES^2,3,4^

^1^Boston University, Boston, MA, ^2^Center for Regenerative Medicine, Boston, MA, ^3^Boston University Chobanian & Avedisian School of Medicine, Boston, MA, ^4^Department of Pediatrics, Boston Medical Center, Boston, MA

**Background/Hypothesis**: Preeclampsia (PE), a placental‐driven pregnancy vascular disorder, has a strong clinical association with risk of bronchopulmonary dysplasia (BPD) and BPD‐associated pulmonary hypertension (BPD‐PH) in premature infants. We hypothesize that PE causes a unique developmental pulmonary vascular injury that predisposes infants to an increased risk of BPD.

**Materials and Methods:** The goal of this study was to identify secreted protein profiles of placental tissues in high risk‐early onset PE patients and test the impact of these secreted placental proteins on human pulmonary endothelial cells. Human placental explants (26‐32 weeks gestation) from PE and preterm (PTL) pregnancies were cultured to generate conditioned media (CM, n = 9 each) which was analyzed using proteomic profiling. PE‐ and PTL‐CM samples were then cultured with human induced pluripotent stem cell‐derived endothelial cells with a lung‐like molecular profile (hiEndo) followed by gene expression analysis.

**Results:** Proteomic analysis identified over 1200 proteins with significant changes in PE‐CM vs PTL‐CM samples. Among these were a unique upregulation of soluble endoglin (sENG) and endothelial protein C receptor (sEPCR), decoy receptors with the potential to block key pulmonary vascular developmental signaling pathways. Gene expression analysis of hiEndo identified that exposure to PE‐CM vs PTL‐CM was associated with significant downregulation in protein secretion pathways and differential expression capillary endothelial cell subtype markers. These included upregulation of *CA4* (Cap2/aCap) and downregulation of *APLNR* (Cap1/gCap).

**Conclusions:** This pilot study has identified a unique profile of secreted decoy receptors from placental tissues in early‐onset human PE. Our results also suggest that endothelial cell exposure to PE placental secreted proteins is associated with alterations in genes regulating central endothelial cell functions and phenotypes. Ongoing studies using these model systems will continue to define pulmonary vascular developmental injury pathways in human PE that could lead to an increased postnatal risk of BPD.

## 13 Experimental Design for Using Mobile Health Intervention and Behavioural Modification to Improve Cardiopulmonary Function and Quality of Life in Pediatric Pulmonary Hypertension

Laguë, S.L.^1,2,*^, Billard, N.^3^, Cakar, A.C.^3^, Annis, S.^3^, Huang S.^4^, Burke K.F.^3^, Strayer T.E.^3^, Becerra, J.^5^, Fineman J.^5^, Jaser, S.S.^6**^, Brittain, E.L.^7**^, Austin, E.D.^8**^

^1^Division of Pediatric Respirology, Department of Pediatrics, BC Children's Hospital, Vancouver, British Columbia, Canada, ^2^Clinical Investigator Program, University of British Columbia, Vancouver, British Columbia, Canada, ^3^Vanderbilt Pulmonary Circulation Center, Vanderbilt University Medical Center, Nashville, Tennessee, ^4^Department of Biostatistics, Vanderbilt University Medical Center, Nashville, Tennessee, ^5^Division of Pediatric Critical Care, Department of Pediatrics, University of San Francisco, San Francisco, California, ^6^Division of Pediatric Psychology, Department of Pediatrics, Vanderbilt University Medical Center, Nashville, Tennessee, ^7^Division of Cardiovascular Medicine, Department of Medicine, Vanderbilt University Medical Center, Nashville, Tennessee, ^8^Division of Allergy, Immunology and Pulmonary Medicine, Department of Pediatrics, Vanderbilt University Medical Center, Nashville, Tennessee

*First author is a pediatric respirology/pulmonology fellow and postdoctoral fellow.

**Co‐senior authors

**Background:** Exercise offers improves functional capacity and quality of life (QoL) in pulmonary hypertension (PH). However, PH can impair cardiopulmonary function, reducing patient QoL. Adult inpatient exercise programs yield substantial benefits, improving health and QoL without medication side effects. Extrapolating these programs into pediatric populations is challenging because large hospital catchments create geographic, financial, and psychosocial barriers. While wearable technologies promote fitness, simply wearing a device doesn't change behavior. When devices are paired with motivational texts through mobile health (mHealth) behavioral coaching, adults with PH have improved activity, cardiopulmonary health, and QoL after 12 weeks.

**Materials and Methods:** Our study pairs Fitbits with mHealth in adolescents. We hypothesize that mHealth tailored to adolescent PH will improve daily step counts, cardiopulmonary fitness, and QoL.

**Results:** In our pilot trial, adolescents with PH at Vanderbilt and UCSF aged 10‐21 years are randomized for 12 weeks to either intervention (mHealth) or control (clinic‐based exercise counseling). Inclusion criteria include WSPH Groups 1 or 4, WHO Functional Class I‐III, and stable PH medications for 3 months. Exclusion criteria include activity restrictions (wheelchair use, walking aids), pregnancy, and no smartphone access. All receive Fitbits to track real‐time activity. The mHealth (intervention) group receives 2‐3 daily automated, personalized texts grounded in behavioral psychology. Daily step count (primary outcome), QoL, and cardiopulmonary function (spirometry, echocardiography, 6‐minute walk, biomarkers, and WHO functional class) are compared between baseline and week 12. We are mid‐recruitment and data collection. 20 patients have enrolled and 5 have completed. ANCOVA models will examine the intervention (mHealth) effect by comparing outcomes at week 12, and adjusting for baseline outcomes and other covariates.

**Conclusions:** This study of remote cardiopulmonary rehabilitation targeting behavioural intervention in pediatric PH may offer scalable, low barrier intervention to improve health and QoL.

**Funding**: NIH NHLBI R34 HL173389 (EDA, SSJ, ELB)

